# Supplementary material for: Orchestrating privacy-protected big data analyses of data from different resources with R and DataSHIELD
Source: PLoS Comput Biol. 2021 Mar 30;17(3):e1008880. doi: 10.1371/journal.pcbi.1008880 (PMC8034722; doi:10.1371/journal.pcbi.1008880)
Supplement: Attachment — Submitted filename: Answer_reviewers.pdf [file pcbi.1008880.s002.pdf]

ISGlobal

Barcelona
Institute for
Global Health

info@isglobal.com
www.isglobal.org

Campus Clínic
Rosselló, 132
08036 Barcelona, Spain
T +34 93 227 1806

Campus Mar
Doctor Aiguader, 88
08003 Barcelona, Spain
T +34 93 214 7300

Dina Schneidman, PhD
Software Editor
PLOS Computational Biology

1st December 2020

Dear Dr. Schneidman,

First of all, we would like to thank you for allowing us to resubmit a revised version of our manuscript entitled "Orchestrating privacy-protected big data analyses of data from different resources with R and DataSHIELD" (former "Orchestrating non-disclosive big data analyses of data from different resources with R and DataSHIELD").

Following your recommendations, we have highlighted in red the changes made in the manuscript and we have also uploaded a clean version. We also enclose a response letter with point-by-point answers to the reviewer comments. We hope that the editor and reviewers can recommend the publication of this new and improved version of the manuscript.

We would also like to address your comment on: "I didn't find a link to the code repository. Please provide it in the revised version". We must say that we created a bookdwon with all the required code. This was stated in the original manuscript as well as in this revised version:
https://isglobal-brge.github.io/resource_bookdown/.

We also want to let you know (also to reviewer #1 and #2) that all the data used in the manuscript is publicly available and also accessible through our Opal demo site as stated in the bookdown:
https://isglobal-brge.github.io/resource_bookdown/opal.html#opal-demo-site and in the manuscript (see comment to reviewer #2). Actually, the reviewer #3 answer yes to this question, maybe because he/she understood how our infrastructure works.

Sincerely yours,

Juan R Gonzalez
Associate Research Professor
Head of Bioinformatic Research Group in Epidemiology
Barcelona Institute for Global Health (ISGlobal)
e-mail: juanr.gonzalez@isglobal.org

A partnership of:
"la Caixa" Foundation
CLÍNIC BARCELONA Hospital Universitari
UNIVERSITAT DE BARCELONA
Generalitat de Catalunya
GOBIERNO DE ESPAÑA
Parc de Salut MAR
Universitat Pompeu Fabra Barcelona
Ajuntament de Barcelona
FUNDACIÓN RAMÓN ARECES

Reviewer #1:

We thank the reviewer for his report on reproducibility. We would like to mention that the aim of this paper is not to provide new biomedical results after analyzing genomic and geographical data. With data analyses we aimed to provide examples of how researchers can analyze data using our proposed infrastructure. This is why the reviewer cannot comment on the reproducibility of our work although as he is mentioning the results in the online book can be perfectly reproduced.

Reviewer #2:

First of all, we would like to thank the reviewer for providing such interesting comments and suggestions that have led manuscript improvement.
Before addressing the reviewer's comments we would like to let him/her know that all of our data are publicly available as it was stated in the bookdown (https://isglobal-brge.github.io/resource_bookdown/opal.html#opal-demo-site). The Opal server also contains the links indicating where they are located (we have added a sentence in page 8 to this regard:

"We have set up an Opal demo site (see Chapter 4 in our bookdown) to illustrate how to perform some basic analyses using DataSHIELD as well as how to deal with different resources for genomic and geographical data. These data are publicly available and can be accessed through DataSHIELD or using the URL available in the Opal site."

Question: Abstract: This abstract is too wordy and uses long sentences. Example: Therefore, big data analyses should ensure appropriate levels of security and privacy, be rigorous with the application of the data confidentiality regulations and address the choice between central data warehousing and the distributed (federated) analysis of data that remain 'in-house'.
Why do we need "therefore"? What is "appropriate"? Why should analyses "address the choice"?

I can understand the basic objective in two sentences: Integrative analysis of multiple large datasets is a common objective in research [here you might focus on computational biology and use that term instead]. When data components of an integrative analysis are managed in distinct and independent systems, steps must be taken to ensure that confidentiality requirements are satisfied at all stages of the analysis.

With one more sentence you could describe key principles and tools to be described in the paper, and the abstract is done. For example, you could mention that DataSHIELD and Obiba are a decade old and that your work enhances these. Note that this reader has never heard of either of these projects, and that there is a cybersecurity company in Arizona USA called Datashield. This could be

confusing, and you may want to clarify that Obiba is an open source project in the domain of bioinformatics, and that DataSHIELD is a collection of R packages usable by users of Opal.

Answer: We thank the reviewer for pointing out this issue. In order to address his/her comment we have modified the abstract by making the sentences shorter and avoiding wording. We also thank the suggestion of how to introduce DataSHIELD and Obiba and have used what the reviewer is indicating. However, we think that using "integrative analysis" may lead to confusion since in computational biology or in bioinformatics this terminology is used to describe how to analyse or combine data from different sources (e.g methylation, gene expression, mutations, …) in a single study. We want to avoid such confusion since our goal is to have a system that integrates the same data from different sources. Therefore, we think that keeping these ideas in the abstract is important.

Action 1: Now the abstract reads:

"Combined analysis of multiple, large datasets is a common objective in the health- and biosciences. Existing methods tend to require researchers to physically bring data together in one place or follow an analysis plan and share results. Developed over the last 10 years, the DataSHIELD platform is a collection of R packages that reduce the challenges of these methods. These include ethico-legal constraints which limit researchers' ability to physically bring data together and the analytical inflexibility associated with conventional approaches to sharing results. The key feature of DataSHIELD is that data from research studies stay on a server at each of the institutions that are responsible for the data. Each institution has control over who can access their data. The platform allows an analyst to pass commands to each server and the analyst receives results that do not disclose the individual-level data of any study participants. DataSHIELD uses Opal which is a data integration system used by epidemiological studies and developed by the OBiBa open source project in the domain of bioinformatics. However, until now the analysis of big data with DataSHIELD has been limited by the storage formats available in Opal and the analysis capabilities available in the DataSHIELD R packages. We present a new architecture ("*resources*") for DataSHIELD and Opal to allow large, complex datasets to be used at their original location, in their original format and with external computing facilities. We provide some real big data analysis examples in genomics and geospatial projects. For genomic data analyses, we also illustrate how to extend the resources concept to address specific big data infrastructures such as GA4GH or EGA, and make use of shell commands. To help researchers use this framework, we describe selected packages and present an online book (https://isglobal-brge.github.io/resource_bookdown)."

Action 2: Additionally, we have re-written some parts in the manuscript to avoid long sentences and going to the point (see red parts in the new version of the manuscript)

Question: Text: The first paragraph repeats much of the abstract; this should be avoided.
Answer: Following reviewer's recommendation, we have avoided repetition and now the first paragraph reads:
Big Data brings new opportunities to biomedicine and challenges to data scientists. These challenges require new computational and statistical paradigms to deal with important principles of data management and data sharing. These are being adopted by an increasing number of studies. The new paradigm should consider: ensuring appropriate levels of security and privacy; the rigorous application of the stringent regulations required by governance frameworks such as GDPR in Europe (https://gdpr-info.eu/) and similar regulatory mechanisms across North America and elsewhere; and a considered choice between central data warehousing and the distributed (federated) analysis of data that remain with their custodian.

Question: The phrase "the Opal data warehouse" is used without explanation. I have never heard of this.
Answer: In order to clarify reviewer's comment we have added this in the abstract:
Opal is a data integration system used by epidemiological studies and developed by the OBiBa open source project in the domain of bioinformatics.

Question: After browsing around obiba and looking at the three figures, I have a sense of what this project is about. The "book" https://isglobal-brge.github.io/resource_bookdown/ is nicely done and is a good supplemental resource for the paper. The figures of the paper give a vague sense of the collections of components in scope, and show "firewalls". But there is no clear depiction of how "non-disclosure" is achieved or guaranteed.
Answer: We thank the reviewer for pointing out this important issue. Actually, it is described in reference 2 (Gaye et al. (2014) DataSHIELD: taking the analysis to the data, not the data to the analysis).
Action: We have added this paragraph in the new version of the manuscript (page 9)

The difference between standard data analysis and that done by DataSHIELD is that the analysis is performed at the location of the data (e.g. the data nodes). No data is transferred from that location, only non-disclosive summary statistics. The set of analytical operations which can be requested to be performed at the location of the data, has been carefully constructed to prevent any attempt for direct or inferential disclosure of any individual-level information. The R parser also blocks any form of arguments that are not allowed in DataSHIELD. For analytical operations which could potential yield results which are disclosive, for example if a small amount of data is being analysed, the operation will check if the results match the data protection policies of the location's data governance rules, before returning any results back to the client (e.g. the analysis node). If any of the protection rules are violated, the client does not receive any results but gets study-side messages with information about potential disclosure issues [2].

Question: The paper suffers from the lack of tables that would help readers to understand capabilities and limitations in comparison to other frameworks.

Answer: We thank the reviewer for providing such recommendation. Actually, we also thought that we lacked tables.
Action: We have created two tables that have been added to the manuscript.
Table 1 describes the features, capabilities/advantages and limitations/disadvantages of our proposed framework (page 18) .
Table 2 provides all the available resources at the resourcer R package and extensions for genomic data (page 21).

Question: The paper also fails to mention GA4GH (Global Alliance for Genomics and Health), that aims to develop standards in this domain. See ga4gh.org, where all the main toolkits would seem to intersect with items described in the paper.
Answer: We thank the reviewer for letting us know this important project. Actually, we think that the existing GA4GH API Bioconductor package could be easily incorporated as a *resource* and use our data analysis methods to exploit GA4GH data. Also, data from EGA (European Genome Archive) could also be a new resource.
Action 1: We have created two new resources for GA4GH and EGA data repositories and extended the use of BAM files for genomic data (see Table 2). This paragraph has been added in page 8:

We have extended the resources available at the resourcer package into different settings. These extensions as well as the current resources that can be accessed through the Opal servers are described in Table 2. So far, we can get data from different locations (Amazon Web Services, HL7 FHIR or Dremio), read other types of files which are specific in genomic studies (BAM, VCF and PLINK) and access data from other infrastructures such as GA4GH, a federated ecosystem for sharing genomic, clinical data (ref #17) and EGA which is a permanent archive that promotes distribution and sharing of genetic and phenotype data consented for specific approved uses. (ref #18)

Action 2: We have also added a new section to the bookdown describing how the resources can be extended to GA4GH and EGA (https://isglobal-brge.github.io/resource_bookdown/extension-to-ga4gh-and-ega-bamvcf-files.html#upload-ga4gh-and-ega-resources-into-opal). This has been mention in the abstract and the results section (subsection “Available resources extensions”)

Question: In summary, the paper reads like a mix between advertising and user manual, and mentions but really does not illuminate connections to computational biology practices. The tools appear well-motivated and well-documented, and may well deserve broad adoption. But this paper as written does not provide a strong argument for engaging with an inevitably complex environment. The basic principles can be articulated simply, but the proof that they are implemented in the system described here, and a demonstration that this implementation should be used by anyone who shares its objectives, will take more work and a more self-critical stance.

Answer: In the revised version, we tried to write the paper in a more scientific way and provide examples of how our proposed framework can help biologists in particular when dealing with genomic data. Having added the resources for GA4GH and EGA as a response to one one the questions made by the reviewer, demonstrate that dealing with this infrastructure is really simple and that can be extended to a wide range of applications in biology as described in Table 2 (Feature: Applications in other biomedical areas than genomics). Nonetheless, deployment can have some limitations and this has been highlighted in Table 2. We would also like to mention that our proposed system is quite simple from a practical point of view as illustrated in our example by analyzing a resource using GA4GH files. We show how performing a principal component analysis (a widely used method in GWAS) from a data which is available in such infrastructure can be really simple using the resources (see https://isglobal-brge.github.io/resource_bookdown/extension-to-ga4gh-and-ega-bamvcf-files.html#descriptive-omic-data-analysis)

Reviewer #3:
We were delighted to read about the datashield resources feature and think it is a great innovation that needs to get large attention from the scientific community. Datashield changes analysis practice by bringing analysis to the data, instead of first centralizing data, and does so in a practical usable way for researchers via the R statistical language. Thus, it provides a solution to the increasing difficult challenge of enabling pooled analysis of data from multiple centers and even countries for sensitive data which has become more challenging because of uncertainties around GDPR etc. However, datashield was limited to only the methods implemented by the datashield team and R who, while doing a great job, cannot be expected to address all analysis needs. The resources approach enables statististicians/bioinformaticians to create bespoke analysis pipelines and distribute them to centers automatically and provide access controls without needing to have much local analyst work (in contrast to sending around a cookbook and then requiring a local statistician to execute by hand).

Answer: We really thank the reviewer for his/her very supportive general comments about our work, and for the suggested comments and suggestions to the manuscript.
Below we have comments and suggestions:

===
Overall we have the following concerns:

Question 1: The manuscript is in places uses many more words then necessary [sic]. This proza sometimes makes it hard to follow. So we would urge the authors to shorten and simplify the text where possible. Also carefully check for typos, in particular on the nice online book.
Answer: We thank the reviewer for his/her careful reading of both the manuscript and manual.

Action: Following reviewer's recommendation we have re-written some parts of the manuscript and removed unnecessary words (see changes in red color in the new version of the manuscript). We have also carefully revised the online book.

Question 2: One of the core features should be that data providers can precisely access control. However, it is not clearly described but based on test we believe it is actually quite simple. We think you should make this explicit from the start, i.e., in abstract, and also deserves a short alinea to describe how it works in practice
Answer: We thank the reviewer for pointing out this important issue. In order to address his/her comment we have mentioned this important point in Table 1 and have added this paragraph to the manuscript (page 10):

We would like to emphasize that with DataSHIELD, analysis is performed at the location of the data. The data provider has full control over what information is transferred from their location to the location of the analyst by setting filters for a number of disclosure traps. This means that the results returned to the analyst can be carefully created to be non-disclosive, and match the policies of the data provider's data governance rules.

Question 3: . While the authors give nice examples on analysis (actually more than needed), one thing that we found missing is how the authors believe complex analysis protocols will be distributed from central analysis site to local data providers. Because in case of sensitive data, we expect data providers to want to limit access to only one resource, i.e. a particular analysis procedure. Then the question is: how will such procedures be distributed from a central analysis site (i.e. lead of a consortium analysis) to all connected sites (i.e. data providers) without needing large local expertise. We believe that for example for distributed meta-analysis such process should be seamless (otherwise still local expertise is needed to operate)
Answer: We thank the reviewer for the positive response about the examples. After writing the new version of the manuscript, we also think that there are more examples analyses than needed. In order to overcome this, we have changed the omic part by considering only genomics. We think this is the best option since after introducing new implementations for GA4GH and EGA repositories the omic examples became huge.
Considering the specific reviewer's question, we think that there is a confusion. The resource is the input of the "analysis procedure" and the "analysis procedure" is available as a R package to be deployed in each data node (thus requiring minimal local expertise). Regarding restricting the use of some "analysis procedure" to some users, it is currently possible only by setting up different Opal/DataSHIELD data nodes. Having different DataSHIELD user profiles (i.e. some "analysis procedure" requiring specific usage permission) is in development for the EUCAN-Connect project.

Question 4: Finally, in our own experience the hosting environment of the participating data providers can be quite heterogeneous. You might want to discuss how you expect to deal with a resource based analysis in for example a

network of 20 data providers and how you would expect the analysis to accommodate these differences (without having to address these all via the central analysis lead).
Answer: This is a very interesting question and even more from a practical point of view. We think that the best option is that all data providers harmonize data in a unique format or hosting environment (the container-based deployment solution is recommended in that regard). We have some experience, for instance, by dealing epigenome data analysis in a set of independent cohorts. As it is described in the bookdown, our analyses can be performed having data encapsulated into ExpressionSet's. What we have done is to send the data providers some functions to create such types of objects and then use our functions implemented in the dsOmics package to analyse data. Another option would be to create specific functions in the server side to put the data from different formats into the required one. But this will require specific developments. The simplest approach is to perform independent analyses at each server and then meta-analyzed the p-values or any other statistic which does not depend on data harmonization.
Action: We have added this sentence to the manuscript (pages 9, 10)

This methodology has some limitations when data are not properly harmonized (e.g. genotyping in different platforms, different VCF versions, ...). In order to overcome this problem, data format validation can also be performed by the analyst using DataSHIELD functions. In genomics, this can be achieved by first doing imputation and then solving issues concerning genomic strand and file format.[23]

===
Some text was hard to follow, and you might want to rephrase
Question 5: ."Such analysis not only demands scalable methods and appropriate mathematical models but must also maintain consistency with fundamental principles of sound data management and data sharing that are common across all areas of health, social and bioscience. Therefore, big data analyses should ensure appropriate levels of security and privacy, be rigorous with the application of the data confidentiality regulations and address the choice between central data warehousing and the distributed (federated) analysis of data that remain 'in-house'."
Answer: We have rephrased the suggested sentences. The reviewer is acknowledge for carefully reading the manuscript

Question 6: . "This requires data generators to physically transfer data [propose you add: to a central analysis server] to make them accessible to analytic users."
Action: following reviewer's recommendation we have rephrase these sentences

Question 7: . I got lost in the Methods section. It would greatly help if you give a short introduction, naming the main elements and how they fit together.
Action: We have addressed this by reordering and editing some of the existing paragraphs. We hope that the section reads better in the current version.

Question 8: . "We define "resource" this data storage or computation access description". I think you want to be more explicit, because apparently 'resource' is a URL that can denote file handle, data access protocol, or execution of some script.
Action: We have rephrased this sentence and now reads

We define a "resource" to be a description of how to access either: (1) data stored and formatted in a particular way or (2) a computation service.

===
Minor suggestions: Furthermore, we have minor suggestions that you can ignore but may be of use to the authors:

Answer: In general, we really appreciate the suggestions (that for us are not so minor) made by the reviewer. Some of them have been addressed and others will be part of our future developments and will be taken into consideration. These are the changes we have included in the new version of the manuscript:

Action 1: Following reviewer's suggestion, we have extended our resources to Dremio and a new R package (https://github.com/obiba/odbc.resourcer) has been created. Related to this we also created other R packages to deal with HL7 FHIR (https://github.com/obiba/fhir.resourcer) and Amazon files (https://github.com/obiba/s3.resourcer). For omic data, we have extended the resources to deal with BAM files (Next Generation Sequencing) and data from GA4GH and EGA that will facilitate federated analyses from genomic repositories (https://isglobal-brge.github.io/resource_bookdown/extension-to-ga4gh-and-ega-bamvcf-files.html).
All this information has been added to Table 2.

Action 2: The reviewer raised several issues related with DataSHIELD features. In order to address these and others, we have created a new table (Table 1, page 18 and 19) describing the main capabilities and limitations of our proposed infrastructure.

Action 3: The chapter 16.3 (Tips and Tricks - current chapter 20) has been finished. Note that now it corresponds to chapter 21 (https://isglobal-brge.github.io/resource_bookdown/tips-and-tricks.html)

Action 4: The reviewer mentioned that Book 16.3 was not finished. The Book has been updated and completed.

Next, we discuss other comments made by the reviewer. We think they are so specific to be included in the manuscript but they can be interesting for some readers. Therefore, we have decided to add a new section to the bookdown

ISGlobal
Barcelona Institute for Global Health
info@isglobal.com
www.isglobal.org
Campus Clínic
Rosselló, 132
08036 Barcelona, Spain
T +34 93 227 1806
Campus Mar
Doctor Aiguader, 88
08003 Barcelona, Spain
T +34 93 214 7300

containing this information (https://isglobal-brge.github.io/resource_bookdown/advancedtechnical-questions.html)

Comment: Resources credentials are fixed and managed by Opal which implies you do not have a refresh token or something like that, that will expire over time. The policy decision and enforcement is now located in the DataSHIELD engine. This means that the resource owner can not decide anymore if someone has access to the resource. Audit logging at the resource side is hard to do this way.
Answer: it is true that a more elaborated authentication/authorization policy cannot be handled on the DataSHIELD server side. Any token must be valid for a programmatic usage, and if it happens to expire or be invalidated, it needs to be renewed and updated in the Opal's definition of the resource. Regarding the auditing, all DataSHIELD user commands are logged by the Opal server. The data owner has both control on the resource data access credentials and the permissions to use this resource.

Comment: If the computational resource needs to do a lot, you need to program that either in the ResourceExtension or as given commands.
When you program an extension you are dependent on the person's choices regarding the interface he/she exposes and when you give it as parameters in the resource you need to parse it in the resourcer package..
For example, how do you prevent malicious SQL injection? We would expect that you would not open up such resources to the external source but only a pre-packaged analysis.
Answer: The DataSHIELD R analysis procedure that makes use of a resource is responsible for interacting in a secure way with the underlying resource system (database or ssh server for instance), like any application that makes use of a database. The best practice is to expose an API that is “business” oriented by defining a limited number of parameters that are easy to validate for each specific operation. This is also facilitated by the DataSHIELD infrastructure built-in feature which consists of allowing a limited syntax for expressing the server side function call parameters.

Comment: You are still bound to the limitations of R in terms of memory and CPU usage when you want to correlate data in R against the data that is available in the resource. You need to either push the data you want to correlate against into the resource or extract it from the resource in the R-environment.
Answer: The resource API offers the possibility to work with the dplyr package which delegates as much as possible the work to the underlying database. More generally, R is only required as an entry point and real data analysis can happen in any system that is accessible by R (for instance ML analysis can be launched from R in a Apache Spark cluster). The DataSHIELD analysis procedures must be programmed in that way, to support large to big datasets analysis. Another improvement that is currently being developed (EUCAN-Connect) is the capability of having multiple R servers for a single DataSHIELD data node, in order to address the case of multiple users accessing concurrently the memory and CPU of a server.

Comment: What is going to happen when you want to finish the analysis on another moment. Or the analysis is taking days how do you retrieve the result.
Answer: A plain R server is probably not the most suitable system for handling long running blocking tasks. Usually this kind of situation is addressed by submitting a task execution request to a worker, which will run the task in the background and will make available the progress of the task and its result for later retrieval. This type of architecture is available in many languages and systems and could be implemented in a DataSHIELD analysis procedure.

Comment: When the analysis is taking a lot of resources in terms of memory and CPU how do you limit this per DataSHIELD-user? Who is responsible for this, DataSHIELD or the resource owner?
Answer: It is possible in R to limit the memory and CPU usage (and much more) using the package RAppArmor. One possible improvement would be the ability to define an AppArmor profile per user or group of users (this profile would be applied at the start of a DataSHIELD session in each data node). The data owner would have the control of the definition of the profiles and which one applies to each user.

Comment: The resource owner needs to implement a way to be easily accessible for the resourcer-package. Especially when you want to run more complex jobs it usually takes a complex interface to work with.
Answer: Building access to a resource is one complexity, that is in practice limited. Allowing multiple/complex parameters for the analysis of a resource is another one, that takes place in the definition of the DataSHIELD R server analysis functions, not in the definition of the resource.

Comment: In the shell and ssh resource the Opal administrator is managing the actions that may be performed by the resource handler (researcher in general). When you host Opal on different infrastructure than the resource and is managed by someone who is not in charge of managing the resource the list of possible commands can be freely added to the resource. This possible allows
Answer: The example of the PLINK resource (i.e. running PLINK through SSH) is put in practice by a using a SSH server in a docker container: the possible actions are limited to the available commands and data in this container for the user that connects to the SSH server (which itself has limited rights). Extra security could be added with setting up an AppArmor constrained environment. This illustrates that security enforcement is possible but needs to be thought ahead of deploying access to such a resource.

Comment: On page 7 and 12 you state that the disclosure control on big data is often more relaxed. In practice we do not encounter this relaxation. We are very keen on analysing data outside our facility but are bound to the current contracts regarding data transfer or access agreements which take a lot of time to arrange. How can we make sure when you offer more open data in a way that it will be legally feasible to access the data without signing a data transfer or large access agreement?

In other words is it practically feasible to do analysis in this way regarding the juridical implications?
Answer: We have omitted this question.

Comment: Maybe less dependencies in the resourcer package?
Answer: We have tried to find a balance between showing out-of-the-box capabilities of various types of resources and not overloading the package. Note that most of the dependencies are suggestions and are not required at installation time. Since the review of the paper, several additional resource extensions have been developed to access less common or more specific resources (Dremio, HL7 FHIR, S3).

Comment: When the interface of a dependency is changed the package needs to be changed as well.
Answer: The resourcer package dependencies are well established packages (DBI, tidyverse etc.) that should not change in the near future. More experimental ones (ODBC, aws.s3) are proposed as additional packages.

Comment: Think of a way to delegate the authorisation and authentication back to the resource owner. We would propose to change the way of passing credentials in the resource.
Answer: The resource owner also owns the resource definition in the Opal server.

Comment: Is it possible to restrict the usage of certain resources? For example, prohibit the use of resources.
Answer: The resource usage requires user/group permission to be set up in Opal. In addition to that, the credentials used to access the resource can be permanently or temporarily invalidated by the resource owner.

Comment: It would be nice to have some way of enforcing the metadata which is tight toi the data to be correct. This is often the problem with longitudinal data that metadata over all columns should be correct. It is likely that this also yields for big data structures that are analysed in a pooled manner. How do you handle multiple versions of VCF for example?
Answer: This is a data harmonization issue that is usually addressed before the analysis. Data format validation, if needed, can also be performed by the analyst using DataSHIELD functions. Considering having different versions of VCF we aim to harmonize data by first doing imputation and solving issues concerning genomic strand and file format (PMID: 25495213). [This issue has also been addressed in the manuscript, pages 9 and 10]
